# The Thai version of the nursing delirium screening scale-Thai: Adaptation and validation study in postoperative patients

**DOI:** 10.3389/fmed.2022.956435

**Published:** 2022-09-23

**Authors:** Pawit Somnuke, Peleen Limprapassorn, Varalak Srinonprasert, Titima Wongviriyawong, Patumporn Suraarunsumrit, Ekkaphop Morkphrom, Unchana Sura-amonrattana, Harisd Phannarus, Duangcheewan Choorerk, Finn M. Radtke, Onuma Chaiwat

**Affiliations:** ^1^Department of Anesthesiology, Faculty of Medicine Siriraj Hospital, Mahidol University, Bangkok, Thailand; ^2^Division of Geriatric Medicine, Department of Medicine, Faculty of Medicine Siriraj Hospital, Mahidol University, Bangkok, Thailand; ^3^Faculty of Medicine, Integrated Perioperative Geriatric Excellent Research Center, Siriraj Hospital, Mahidol University, Bangkok, Thailand; ^4^Department of Preventive and Social Medicine, Faculty of Medicine Siriraj Hospital, Mahidol University, Bangkok, Thailand; ^5^Department of Nursing, Faculty of Medicine Siriraj Hospital, Mahidol University, Bangkok, Thailand; ^6^Department of Anaesthesia and Intensive Care, Nykoebing Hospital, University of Southern Denmark (SDU), Odense, Denmark

**Keywords:** delirium, DSM-5, Nu-DESC, postoperative, screening test

## Abstract

**Background:**

The Nursing Delirium Screening Scale (Nu-DESC) is an effective instrument for assessing postoperative delirium (POD). This study translated the Nu-DESC into Thai (“Nu-DESC-Thai”), validated it, and compared its accuracy with the Diagnostic and Statistical Manual of Mental Disorders-5 (DSM-5).

**Methods:**

The translation process followed the International Society for Pharmacoeconomics Outcome Research guidelines. Recruited participants were ≥ 70 years old, fluent in Thai, and scheduled for surgery. The exclusion criteria were cancellation or postponement of an operation, severe visual or auditory impairment, and patients with a Richmond Agitation Sedation Scale score of –4 or less before delirium assessment. Post-anesthesia care unit (PACU) nurses and residents on wards each used the Nu-DESC to assess delirium in 70 participants (i.e., 140 assessments) after the operation and after patient arrival at wards, respectively. Geriatricians confirmed the diagnoses using video observations and direct patient contact.

**Results:**

The participants’ mean age was 76.5 ± 4.6 years. The sensitivity and specificity of the Nu-DESC-Thai at a threshold of ≥ 2 were 55% (95% CI, 31.5–76.9%) and 90.8% (84.2–95.3%), respectively, with an area under a receiver operating characteristic curve (AUC) of 0.73. At a threshold of ≥ 1, the sensitivity and specificity were 85% (62.1–96.8%) and 71.7% (62.7–79.5%), respectively (AUC, 0.78). Adding 1 point for failing backward-digit counting (30–1) to the Nu-DESC-Thai and screening at a threshold of ≥ 2 increased its sensitivity to 85% (62.1–96.8%) with the same specificity of 90.8% (84.2–95.3%).

**Conclusion:**

The Nu-DESC-Thai showed good validity and reliability for postoperative use. Its sensitivity was inadequate at a cutoff ≥ 2. However, the sensitivity improved when the threshold was ≥ 1 or with the addition of backward counting to Nu-DESC-Thai and screening at a threshold of ≥ 2.

## Introduction

Delirium is one of the most unwanted conditions in individuals > 65 years of age. The condition is highly prevalent among older patients with baseline mild or major neurocognitive disorders, those on mechanical ventilation, and postoperative older patients undergoing surgery with general anesthesia (1, 2). In the general population, the incidence of delirium has been reported to be between 11 and 32% in hospitalized patients and more than 80% among critically ill patients admitted to intensive care units (ICUs) (3–6). In addition, over 50% of non-cardiac surgical patients were reported to have delirium beginning from postoperative day 1 and beyond (7). Postoperative delirium (POD) can develop in the post-anesthesia care unit (PACU), where the incidence has been reported to range from 10 to 20% (8–12). Without proper prevention and detection, delirium increases hospital costs and results in higher infection rates, such as pneumonia, urinary tract infections, and local wound infections. In addition, deterioration of quality of life, prolonged hospital stays, additional burdens on caregivers, increased mortality and post-hospital discharge cognitive and functional decline are established consequences (13–16).

Several tools have been developed to detect delirium. The gold standard for diagnosing delirium is the clearly defined criteria detailed in the Diagnostic and Statistical Manual of Mental Disorders, Fifth Edition (DSM-5) (17, 18). DSM-5 had been proofed with good validity, reliability and accuracy against DSM-IV with sensitivity 100% and specificity 98% (19). Additionally, DSM-5 was capable of detecting severe delirious cases with high risk of mortality (20). However, DSM-5 assessments should be conducted by specially trained experts, such as a psychiatrist, a neurologist, or a geriatrician (18). Other assessment tools that are more feasible to use include the Confusion Assessment Method for the ICU (CAM-ICU), the 4 “A”s test (4AT; Arousal, Attention, Abbreviated Mental Test-4, Acute change), and the Nursing Delirium Screening Scale (Nu-DESC). The CAM-ICU is the most frequently used tool for ICU patients (21–23). The 4AT is also a rapid screening tool for delirium with high sensitivity and specificity among older hospitalized patients. Both tools have been translated into Thai.

The Nu-DESC is a simple, practical, and time-saving instrument for detecting patients at risk of delirium outside the ICU. With its high sensitivity and specificity, the test was introduced by Gaudreau et al. for patients with hemato-oncological disorders (24). Nurses can perform the test in 1–2 min (24) in conjunction with routine patient care in various settings, including wards and postoperative holding areas. Its feasibility and practicality have led to its translation into multiple languages and validation against the DSM-IV or DSM-5 in several countries (24, 25). As an evidence-based and consensus-based guideline, the Nu-DESC is recommended by the European Society of Anesthesiology for the POD complication prevention and monitoring (26). However, it has not been translated into Thai or applied in clinical practice in Thailand.

Given the simplicity and reliability of the Nu-DESC, it is very valuable in postoperative settings both inside and outside the ICU. However, it needs to be translated and validated into a Thai version. This will ensure its reliability and accuracy for the early detection of delirium and its management in Thailand. The primary objective of this study was:

• To translate the original Nu-DESC into a Thai version (“Nu-DESC-Thai”) following the guidelines of the International Society for Pharmacoeconomics and Outcome Research (27).

The secondary objectives were

• To validate the Nu-DESC-Thai against the DSM-5 for use as a screening tool for delirium in postoperative patients in PACUs and wards and
• To determine an increase in Nu-DESC-Thai sensitivity when the attention test was included.

## Materials and methods

### Study design

We conducted a blinded cross-sectional study of POD assessments. Nurses in the PACU and resident anesthesiologists on wards and the ICU used the Nu-DESC-Thai to perform the delirium screening. The screening was compared with the DSM-5-based diagnoses determined by board-certified geriatricians.

### Setting and procedure

The study was conducted at a university hospital after being approved by the Institutional Review Board (IRB), Faculty of Medicine Siriraj Hospital, Mahidol University (Si 697/2019). The data were collected from August 2020 to March 2021. Our study comprised 2 phases.

#### Phase 1: Translation process of nursing delirium screening scale-Thai from original English version

The translation of the Nu-DESC from English to Thai followed the International Society for Pharmacoeconomics and Outcome Research guidelines, as described previously (28). Briefly, after receiving permission from the original author (24), the process involved (1) forward translation by 2 independent physicians, (2) reconciliation to a single forward translation, (3) independent back-translation to English by 2 American-board-certified Thai anesthesiologists blinded to the original English version, and (4) review of the back-translation. The English back-translation was then sent for examination by the original author to ensure the accuracy of the content.

Cognitive debriefing was subsequently performed by experienced recovery room nurses and doctors from different clinical specialties (Supplementary Tables 1, 2). The debriefing was reviewed and corrected. The 2 initial forward translators proofread the final version of the Nu-DESC-Thai. The Nu-DESC-Thai was content validated before use by 3 experts including 2 geriatricians and a psychiatrist. Each item of Nu-DESC-Thai was assessed and scored by the experts as 1, 0, or –1 for agree, no idea or disagree, respectively. An index of item-objective congruence of 0.74 was used to assess the validity of the Nu-DESC-Thai (Supplementary Table 3). Inter-rater reliabilities were tested with the ratings given by a certified Nu-DESC assessor. The reliability coefficients of a trained resident anesthesiologist and 10 PACU nurses were 100 and 98.18%, respectively (Supplementary Tables 4, 5). Based on its acceptable content validity and inter-rater reliability, the Nu-DESC-Thai was ready for clinical use (Supplementary Table 6).

#### Phase 2: Validation of nursing delirium screening scale-Thai delirium screening performance against diagnostic and statistical manual-5 reference standard

Recruitment was conducted among patients at least 70 years old, fluent in Thai, and scheduled to undergo elective, moderate to major surgery. Informed consent to participate in the trial was obtained upon admission to a ward the day before the scheduled operation. The reasons for exclusion were cancellation or postponement of an operation for proper preoperative patient optimization, severe visual or auditory impairment, and patients with a Richmond Agitation Sedation Scale score of –4 or less before the proposed delirium screening assessment.

### Assessment tools

The participants were evaluated throughout the course of the study by the following assessment tools:

#### Nursing delirium screening scale-Thai

The Nu-DESC-Thai assessed 5 items including disorientation, inappropriate behavior, inappropriate communication, illusions/hallucinations and psychomotor retardation.

Each item was scored according to severity according to severity as 0, 1 and 2 for no symptom, mild symptom and severe symptom, respectively. According to the original English version of the Nu-DESC, delirium was characterized by a total score of ≥ 2 (24).

#### Diagnostic and statistical manual-5 reference gold standard

Board-certified geriatricians performed delirium diagnoses by applying the following DSM-5 criteria (16, 17).

#### Nursing delirium screening scale with backward-digit counting

Because the Nu-DESC is based on behavioral observations, it does not consider the attention domain (25). Therefore, the participants were assessed using an attention test requiring them to count backwards from 30 to 1. Backward counting indicates an intact working memory and the ability to concentrate and maintain attention. Additionally, the backward counting test was easy for patients to comprehend and assessors to interpret (29). Any counting errors yielded a total score of 1, whereas all correct counting would score 0. The final assessment threshold was determined after adding the Nu-DESC-Thai scores with backward counting.

### Screening methods

The baseline clinical evaluations of the patients were performed preoperatively through a general interview to determine cognition, orientation, and attention. The interviews were carried out by a third-year anesthesiologist trainee on the ward the day before the operation. The resident anesthesiologist was trained in conducting delirium assessments using the Nu-DESC-Thai. Geriatricians reviewed the recorded interviews. Doing so enabled the identification of any changes between the baseline cognitive status and subsequent postoperative assessments in the PACU and wards.

The geriatricians suggested integrating questions and attention tests (backward counting from 30 to 1) into the Nu-DESC-Thai screening to assess memory, attention, and communication. The questions were birthdate, birthplace, and reason for admission to the hospital. However, these questions were only meant to provide the geriatricians with a general overview of patients’ cognitive status. They were not intended to interfere with the interpretation of the delirium screening by the Nu-DESC-Thai.

The PACU nurses performed the first delirium screenings using the Nu-DESC-Thai approximately 45 min to 1 h after the end of the operation and prior to the transfer of the patient back to a ward. Critical patients who needed close clinical observation were transferred to the ICU, where a trained anesthesiology resident assessed them for POD 45 min to 1 h after the operation. Because of the rapid turnover of patients in the PACU and the crowded operating room environment, geriatricians would have had difficulty visiting patients in the PACU in time to assess delirium using the DSM-5. Consequently, the PACU nurses and the trained resident recorded the videos of the Nu-DESC-Thai delirium screenings in the PACU and ICU. Blinded to the Nu-DESC-Thai assessment scores, the geriatricians compared the recorded preoperative and postoperative Nu-DESC screenings. They then diagnosed delirium using the DSM-5 criteria.

**FIGURE 1:**
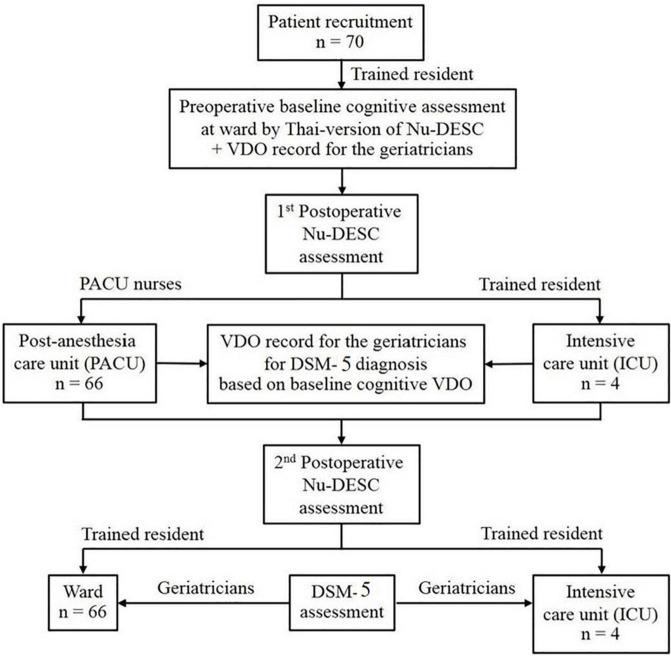
Consort flow of the Nu-DESC assessments. ICU, Intensive Care Unit, Nu-DESC, Nursing Delirium Screening Scale, PACU, Post-Anesthesia Care Unit.

The second appraisals of delirium were completed in a ward or the ICU within 24 h after the operation. During the second evaluations, the trained resident and the geriatricians independently determined delirium through a direct patient approach using the Nu-DESC-Thai and the DSM-5, respectively (Figure 1). The geriatricians assessed delirium through patient and caregiver/family member interview as well as clinical evaluation and patient chart review.

### Sample size calculation and participants

According to Gaudreau et al., the original English version of the Nu-DESC has a sensitivity of 85.7*%* and a specificity of 86.6% (24). Based on a sensitivity of 0.8 and an error of 0.20, the total number of cases of delirium was calculated as 16. Given that the delirium incidence is approximately 15% for postoperative surgical patients (1, 4–6), 106 assessments were needed. To account for a 20% loss of data for any reason, the sample size was increased to 132 evaluations. However, in this study delirium for each patient was assessed twice (i.e., 1 h after surgery at PACU and 24 h after surgery at wards) due to possible fluctuation of delirium symptoms. Therefore, 70 patients were recruited to get 140 POD assessments.

### Statistical analysis

The demographic characteristics of the patients are presented as mean ± SD, percentage, or median (interquartile range). The incidence of POD determined by the Nu-DESC-Thai or the DSM-5 is presented as a percentage. The proportion of patients meeting each of the 5 items assessed by the Nu-DESC-Thai was reported as a percentage. The diagnostic performance of the Nu-DESC-Thai was determined in terms of sensitivity and specificity. The positive predictive value (PPV) and negative predictive value (NPV) were reported by the 95% CI. Receiver operating characteristics were adopted for the optimal threshold analysis of the Nu-DESC-Thai relative to the DSM-5. The area under the receiver operating characteristic curve (AUC) determined the overall test performance. An AUC of 0.5 represented no discriminative ability, whereas an AUC of 1.0 indicated excellent discrimination (30). Statistical analyses were performed with SPSS Statistics for Windows, version 18.0 (SPSS Inc., Chicago, IL, USA).

## Results

A total of 70 patients were recruited for the study. Their mean age was 76.5 ± 4.6 years (mean ± SD), and the majority were women (65.7%). A third (31.4%) of the patients had pre-existing cognitive impairment (indicated by a score ≥ 3.42 for the modified Informant Questionnaire on Cognitive Decline in the Elderly) (31). A small proportion (14.3%) had limited daily activities with dependency on essential personal care (determined by Barthel activities of daily living scores of ≤ 70) (32). Regarding their American Society of Anesthesiologists (ASA) physical status, 47% had ASA class II (a patients with mild systemic disease without substantive functional limitations for example; well controlled type 2 diabetes mellitus, hypertension, dyslipidemia or asthma) (33), and 53% had class III (a patients with severe systemic disease with substantive functional limitations for example; poorly controlled type 2 diabetes mellitus, hypertension, coronary artery disease or cerebrovascular accident) (33). More than 80% of the patients presented with hypertension. All patients underwent an elective procedure with an average anesthesia time of 191 ± 86.5 min. Most of the patients (94.3%) were transferred postoperatively to the PACU. After 1 h of observation in the PACU, they were transferred to a ward. A small proportion of patients who needed critical management and close monitoring (5.7%) were transferred directly to the ICU. All patients were extubated and fully awake before being transferred from the operating room (Table 1).

**TABLE 1:** Demographic characteristics of the patients.

Variables	*n* = 70
**Age** (years)	76.5 ± 4.6
**Female**	46 (65.7%)
**Modified IQCODE score ≥ 3.42**	22 (31.4%)
**BADL score ≤ 70**	10 (14.3%)
**Comorbidities**	
Hypertension	57 (81.4%)
Dyslipidemia	34 (48.6%)
Diabetes mellitus	32 (45.7%)
Chronic kidney disease	8 (11.4%)
Stroke (full recovery)	4 (5.7%)
**ASA classification**	
2	33 (47.1%)
3	37 (52.9%)
**Physical limitation**	
None	51 (72.9%)
Visual	2 (2.9%)
Mobilization	17 (24.3%)
**Site of surgery**	
Abdomen	27 (38.6%)
Vascular	2 (2.9%)
Orthopedic	33 (47.1%)
Head and neck	8 (11.4%)
**Operation time (min)**	139.7 ± 75.8
**Anesthesia time (min)**	191.0 ± 86.5
**Anesthesia type**	
General	52 (74.3%)
Regional	16 (22.9%)
Combined	2 (2.9%)
**Total fluid intake (ml)**	900 (588–1,500)
**Intraoperative blood loss (ml)**	50 (20–250)
**Patient transfer**	
ICU	4 (5.7%)
Ward	66 (94.3%)

Data presented as mean ± SD, *n* (%) or median (interquartile range: IQR). ASA, American Society of Anesthesiologists; BADL, Barthel activities of daily living; ICU, Intensive Care Unit; IQR, interquartile range; Modified IQCODE, Modified Informant Questionnaire on Cognitive Decline in the Elderly; Nu-DESC, Nursing Delirium Screening Scale; PACU, Post-Anesthesia Care Unit.

Table 2 lists the proportion of patients with screening thresholds of ≥ 2 and ≥ 1 who met each of the 5 items assessed by the Nu-DESC-Thai at the first and second assessments. In over 80% of the delirium-positive cases, disorientation was present. Using the DSM-5 as the reference standard for detecting delirium, the sensitivity and NPV of the Nu-DESC-Thai with a cut point of ≥ 2 at the first and second assessments were noticeably lower than when the threshold was reduced to ≥ 1. The opposite was observed for the specificity and PPV of the test. Similar patterns were observed with the total Nu-DESC-Thai assessments (first and second assessments combined; Table 3). Interestingly, when the backward counting test was applied to the Nu-DESC-Thai assessments, the sensitivity, PPV, and NPV significantly improved. The optimal screening values of the total Nu-DESC-Thai assessments were achieved when the backward counting test was added to the Nu-DESC-Thai and the threshold of ≥ 2 was used (sensitivity, 85.0%; specificity, 90.8%; PPV, 60.7%; and NPV, 97.3%). The AUC for the Nu-DESC-Thai with backward counting at a threshold of ≥ 2 was 0.88 (95% CI, 0.78–0.97; Table 3).

**TABLE 2:** Number and percentage of delirious patients categorized by each item of the Thai-version of the Nu-DESC.

Items		Positive delirium cases assessed by Nu-DESC					

		**1^st^ assessment in PACU or ICU**		**2^nd^ assessment in ward or ICU**		**Total assessments**	
	**Threshold**	**≥2 (*n* = 17)**	**≥1 (*n* = 32)**	**≥2 (*n* = 5)**	**≥1 (*n* = 19)**	**≥2 (*n* = 22)**	**≥1 (*n* = 51)**
Disorientation		14 (82.4%)	26 (81.3%)	5 (100%)	19 (100%)	19 (86.4%)	45 (88.2%)
Inappropriate behavior		5 (29.4%)	6 (18.8%)	0	0	5 (22.7%)	6 (11.8%)
Inappropriate communication		8 (47.1%)	8 (25.0%)	3 (60%)	3 (15.8%)	11 (50%)	11 (21.6%)
Illusions/hallucinations		5 (29.4%)	5 (15.6%)	0	0	5 (22.7%)	5 (9.8%)
Psychomotor retardation		13 (76.5%)	15 (46.9%)	1 (20%)	1 (5.3%)	14 (63.6%)	16 (31.4%)

Data presented as *n* (%). ICU, Intensive Care Unit; Nu-DESC, Nursing Delirium Screening Scale; PACU, Post-Anesthesia Care Unit.

**TABLE 3:** Delirium screening performance of the Thai-version of the Nu-DESC compared with the DSM-5 standard reference.

Assessments	Diagnostic threshold	% sensitivity (95% CI)	% specificity (95% CI)	PPV (95% CI)	NPV (95% CI)	AUC (95% CI)
**1^st^ in PACU or ICU (*n* = 70)**	Threshold ≥ 2	61.5% (31.6–86.1)	84.2% (72.1–92.5)	47.1% (29.8–65.0)	90.6% (82.7–95.1)	0.73 (0.56–0.90)
	Threshold ≥ 2 with backward counting	84.6% (54.6–98.1)	81.2% (72.1–92.5)	55.0% (39.1–69.9)	96.0% (87.0–98.9)	0.84 (0.72–0.97)
	Threshold ≥ 1	84.6% (54.6–98.1)	63.2% (49.3–75.6)	34.4% (25.8–44.2)	94.7% (83.2–98.5)	0.74 (0.60–0.88)
	Threshold ≥ 1 with backward counting	100% (75.3–100)	61.4% (45.6–74.0)	37.1% (29.9–45.1)	100%–	0.81 (0.71–0.91)
**2^nd^ in ward or ICU (*n* = 70)**	Threshold ≥ 2	42.9% (9.9–81.6)	96.8% (89.0–99.6)	60.0% (23.1–88.2)	93.9% (88.9–96.7)	0.70 (0.45–0.95)
	Threshold ≥ 2 with backward counting	85.7% (42.1–99.6)	96.8% (89.0–99.6)	75.0% (42.6–92.4)	98.4% (90.9–99.7)	0.91 (0.76–1.00)
	Threshold ≥ 1	85.7% (42.1–99.6)	79.4% (67.3–88.5)	31.6% (20.7–45.0)	98.0% (89.0–99.7)	0.83 (0.66–0.99)
	Threshold ≥ 1 with backward counting	100% (59.0–100)	77.8% (65.5–87.3)	33.3% (24.0–44.3)	100%–	0.89 (0.81–0.97)
**Total (*n* = 140)**	Threshold ≥ 2	55.0% (31.5–76.9)	90.8% (84.2–95.3)	50.0% (33.4–66.6)	92.4% (88.2–95.2)	0.73 (0.59–0.87)
	Threshold ≥ 2 with backward counting	85.0% (62.1–96.8)	90.8% (84.2–95.3)	60.7% (46.1–73.7)	97.3% (92.7–99.0)	0.88 (0.78–0.97)
	Threshold ≥ 1	85.0% (62.1–96.8)	71.7% (62.7–79.5)	33.3% (26.3–41.2)	96.6% (90.9–98.8)	0.78 (0.68–0.89)
	Threshold ≥ 1 with backward counting	100% (83.2–100)	70.0% (61.0–78.0)	35.7% (29.7–42.2)	100%–	0.85 (0.79–0.91)

Data presented as median (95% confidence interval). AUC, area under receiver operating characteristic (ROC) curve; CI, confidence interval; ICU, Intensive Care Unit; Nu-DESC, Nursing Delirium Screening Scale; PACU, Post-Anesthesia Care Unit; PPV, positive predictive value; NPV, negative predictive value.

**FIGURE 2:**
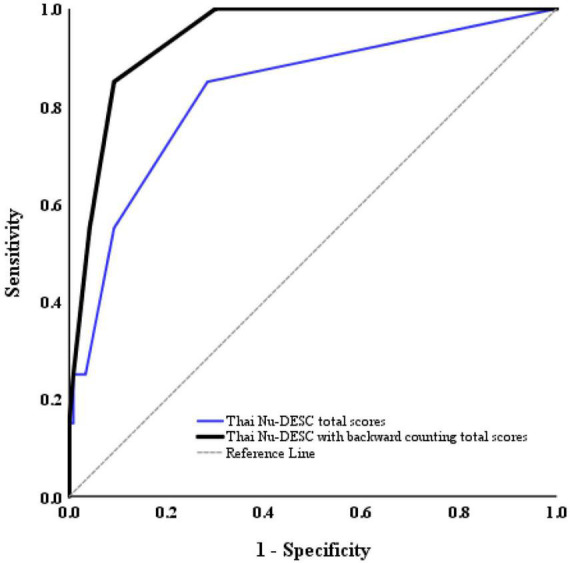
ROC curve for the Thai Nu-DESC total scores (blue line, AUROC = 0.83) and Thai Nu-DESC with backward counting total scores (black line, AUROC = 0.94).

The Nu-DESC-Thai was used as a delirium screening tool at the first assessment (in the PACU or ICU) and the second assessment (in a ward or the ICU). The AUC analysis proved that, compared with the DSM-5, the Nu-DESC-Thai could discriminate between delirious and non-delirious patients. An AUC value of 0.83 (95% CI, 0.72–0.93) was achieved for the Nu-DESC-Thai. With the inclusion of backward counting, the Nu-DESC-Thai achieved a AUROC value of 0.94 (95% CI, 0.90–0.98), signifying a very high screening accuracy (30) (Figure 2).

## Discussion

The most important result of our investigation is that the Thai version of the Nu-DESC is a reliable and valid test. With a screening threshold of ≥ 2, the original study on the English version of the Nu-DESC demonstrated high specificity (86.6%) and sensitivity (85.7%) (24). By comparison, our study demonstrated that the Nu-DESC-Thai had a sensitivity of 55% and a specificity of 90.8%. After adding the backward counting test, the sensitivity of the Nu-DESC-Thai rose to 85% and even 100% when the screening threshold was reduced to ≥ 1.

Although the CAM-ICU has a higher specificity (100%) than the Nu-DESC-Thai and a similar sensitivity (83%), it takes longer to complete (34). Furthermore, the sensitivity of the CAM-ICU was found to fall to 66.7% when used by non-specialized healthcare personnel (35). Another tool, the Clinical Assessment of Confusion (CAC), also has low sensitivity (36%), which means that it might not be the best option (36). A further helpful tool for delirium screening is the 4AT. It is a standard in clinical practice because it is short and straightforward to administer (37). However, studies on the application of 4AT in various clinical settings and its performance in patients with pre-existing cognitive dysfunction are limited (38). Nurses are the key to evaluate delirium because they spend more time closely observing patients than other medical professionals (39). The Nu-DESC is user-friendly, less time-consuming than most alternatives, specifically designed for use by nurses, beneficial, and able to be used in ward and postoperative settings.

It should be noted that although the DSM-5 provides the standard diagnostic criteria for delirium, it is long and requires the help of specialists (18). A test that offers similar accuracy to the DSM-5 in detecting delirium would be valuable in busy clinical settings. Only a few studies have validated the Nu-DESC against the DSM-5 among hospitalized patients in wards or the ICU. One study characterized the Nu-DESC as a test with high specificity (98%) but relatively low sensitivity (42%) compared with the DSM-5 (25). Another study reported a stronger correlation between the Nu-DESC and the DSM-5, with a sensitivity of 92% and a specificity of 95.2% (40).

Unlike most previous studies that assessed hospitalized patients, we selected the Nu-DESC as an optimal tool for delirium detection and explored its usability in postoperative patients within 1–24 h after patients transfer from the operating room. Specifically, we sought to develop a screening test with high accuracy and ease of use, especially for PACU and ward nurses in a postoperative setting.

The Nu-DESC was previously proven effective for routine POD screening (using the DSM-IV as a reference standard). In the PACU, the Nu-DESC had a sensitivity of 95% and a specificity of 87% (10). In wards, its sensitivity was 98%, while its specificity was 92% (41). The study by Neufeld et al. revealed similar specificity but lower sensitivity (32% in the PACU and 29% in wards). However, reducing the Nu-DESC screening threshold from ≥ 2 to ≥ 1 dramatically improved sensitivity: 80% for the PACU and 72% for wards (42). We speculated that the test’s low sensitivity could be due to the nature of the Nu-DESC, which lacks attention and cognition tests.

Our findings reported comparable results to previous studies that compared the Nu-DESC with the DSM-IV and DSM-5 (25, 42). The Nu-DESC at a threshold of ≥ 2 had a low screening sensitivity. On the other hand, the threshold of ≥ 1 increased the sensitivity, but the specificity declined. When utilized with the Nu-DESC, a memory and attention test improved sensitivity, as suggested in the preceding report (25). We incorporated memory and attention appraisal in the current work by adding the backward-digit counting test to the Nu-DESC-Thai. This test is linguistically comprehensible throughout Thailand. We did not adopt the month-backward test for attention assessment because of the diversity of regional dialects used by the Thai population.

The sensitivity improved as anticipated. Optimal Nu-DESC-Thai results were obtained with the inclusion of backward counting and a threshold of ≥ 2, giving a sensitivity of 85% and a specificity of 90.8%. The sensitivity reached 100% with a threshold of ≥ 1 and backward counting. However, the false-positive rate (30%) was much higher than that with a threshold of ≥ 2 (9.2%). We therefore recommend using a threshold of ≥ 2 as the Nu-DESC cutoff coupled with backward counting to detect delirium in postoperative patients. As proposed by previous study, Nu-DESC lacked the key feature for delirium detection which was the ability to recognize abrupt cognitive change from baseline (25). Although adding the backward counting to the Nu-DESC-Thai might unsurprisingly improve the test sensitivity, it was essential to enhance the screening power of Nu-DESC.

Our study demonstrated many strengths. We carefully designed its methodology to allow the trained assessors (the PACU nurses and the resident on wards) to independently evaluate the patients within a short time of each other. This approach eliminated the interpretation discrepancies that arise from the nature of delirium, which fluctuates over time. The participants in our study also underwent diverse types of surgeries and anesthetic techniques, thus ensuring generalizability among the study population. Furthermore, our results demonstrated the application of Nu-DESC-Thai to non-intubated postoperative patients in the ICU. This finding suggests that the Nu-DESC is a versatile screening tool for many clinical settings.

There were still several limitations in our study. First, nurses performed the Nu-DESC screenings in the PACU, whereas trained residents performed subsequent assessments in wards. These assessors were briefly trained how to score the Nu-DESC-Thai before it was adopted in postoperative patients. Also, there can be discrepancies in delirium screening between different groups of raters (43). Nevertheless, our Nu-DESC-Thai achieved excellent interrater reliability (Supplementary Tables 4, 5). A continuing Nu-DESC-Thai training education program would help increase the test accuracy for future use.

Second, the delirium diagnoses in the PACU were based on video records. In contrast, the diagnoses in wards drew upon patient and caregiver interviews to determine whether there had been a change in mental status from baseline. The differing approaches were necessary because the different work schedules of the geriatricians and the PACU nurses limited the opportunities for the geriatricians to perform direct patient evaluations in the PACU.

Third, this study contained a low incidence of POD (20 out of 140 assessments (14.3%) according to the DSM-5 criteria), and it was conducted using a single-center hospital cohort. Although the incidence was similar to those of previous reports (11–13), it is highly desirable to further explore the benefits of the Nu-DESC in a larger population. Future prospective studies are needed to gain more insight on delirium in postoperative as well as general hospitalized patients. With the Nu-DESC-Thai implementation to routine practice, the more rapid screening of delirium is expected. Furthermore, the complications from late detection of delirium including prolonged hospital stay and increased mortality could be minimized.

## Conclusion

This study developed a Thai version of the Nu-DESC. The tool’s suitability for screening delirium in postoperative patients was subsequently successfully established by comparing it with the DSM-5 as the reference standard. Furthermore, Nu-DESC-Thai achieved higher test sensitivity comparable to the original English version after adding the backward counting test. Therefore, it can be routinely applied as an effective delirium screening tool for postoperative patients in the PACU and wards in order to prevent complications related to delirium.

## Data availability statement

The original contributions presented in this study are included in the article/Supplementary material, further inquiries can be directed to the corresponding author.

## Ethics statement

All patients in the study agreed to participate and were informed about the study orally and in writing. The participants returned informed consent forms prior to their participation. This study was performed with the prior approval of the Institutional Review Board, Faculty of Medicine Siriraj Hospital, Mahidol University (Si 697/2019). The patients/participants provided their written informed consent to participate in this study.

## Author contributions

PSo and OC were responsible for the study conceptualization, data analysis, manuscript, preparation, and manuscript writing. PL, TW, PSu, EM, US-A, and HP contributed to the data collection. VS contributed to the study design and manuscript review. All authors read and approved the final manuscript.
