# Supplementary material for: The Thai version of the nursing delirium screening scale-Thai: Adaptation and validation study in postoperative patients
Source: Front Med (Lausanne). 2022 Sep 23;9:956435. doi: 10.3389/fmed.2022.956435 (PMC9537571; doi:10.3389/fmed.2022.956435)
Supplement: Supplementary file 1 [file Data_Sheet_1.PDF]

Supplementary Material

Table 1 Characteristics of the participating staff on the cognitive debriefing

**Characteristics**	**n = 17**
Sex	
Male	3 (17.6%)
Female	14 (82.4%)
Profession	
Doctor	7 (41.2%)
Nurse	10 (58.8%)
Workplace	
Department of Anesthesiology	4 (23.5%)
Department of Geriatric Medicine	2 (11.8%)
Department of Psychiatry	1 (5.9%)
Recovery room	10 (58.8%)

Data presented as n (%)

Table 2 Cognitive debriefing of the Thai-version of the Nu-DESC

Items	Criteria		Professional group: Doctors (n = 7)	Professional group: Nurses (n = 10)	*P* value
1. Disorientation	Understanding	Language	4.14 ± 0.90	4.40 ± 0.52	0.465
		Content	4.43 ± 0.98	4.50 ± 0.53	0.864
	Feasibility	Time	4.43 ± 0.79	4.10 ± 0.74	0.401
		Usability	4.43 ± 0.79	4.20 ± 0.63	0.537
2. Inappropriate behavior	Understanding	Language	4.86 ± 0.38	4.50 ± 0.53	0.125
		Content	4.86 ± 0.38	4.50 ± 0.53	0.125
	Feasibility	Time	4.57 ± 0.53	4.20 ± 0.63	0.225
		Usability	4.57 ± 0.53	4.40 ± 0.52	0.517
3. Inappropriate communication	Understanding	Language	4.43 ± 0.79	4.30 ± 0.48	0.709
		Content	4.43 ± 0.79	4.40 ± 0.52	0.929
	Feasibility	Time	4.00 ± 1.41	4.20 ± 0.63	0.696
		Usability	4.14 ± 1.46	4.40 ± 0.52	0.612
4. Illusions/Hallucinations	Understanding	Language	3.57 ± 1.27	4.40 ± 0.52	0.081
		Content	3.86 ± 1.35	4.30 ± 0.67	0.382
	Feasibility	Time	3.86 ± 1.35	4.30 ± 0.67	0.382
		Usability	3.57 ± 1.51	4.40 ± 0.52	0.126
5. Psychomotor retardation	Understanding	Language	4.29 ± 0.95	4.30 ± 0.48	0.972
		Content	4.14 ± 1.46	4.40 ± 0.52	0.612
	Feasibility	Time	4.43 ± 0.53	4.20 ± 0.63	0.448
		Usability	4.43 ± 0.53	4.40 ± 0.52	0.913

Assessment of the test items according to understanding and feasibility. Rating was determined on a 6-step Likert scale (5, strongly agree; 4, agree; 3, slightly agree; 2, slightly disagree; 1, disagree; 0, strongly disagree). Data presented as mean ± SD of the two groups with statistical significance at $P < 0.05$

Table 3 Content validity testing of the Thai-version of the Nu-DESC

Items	Content expert			ΣR	IOC = ΣR/N
	1	2	3		
Disorientation	1	1	1	3	1.00
Inappropriate behavior	0	1	1	2	0.67
Inappropriate communication	1	1	0	2	0.67
Illusions/Hallucinations	1	1	0	2	0.67
Psychomotor retardation	0	1	1	2	0.67
				**Mean**	**0.74**

Scoring codes: 1 = agree; 0 = no idea; -1 = disagree
IOC, Item-objective congruence

Table 4 Interrater reliability testing between the certified assessor and the trained anesthesiology resident

Patient	Number of errors	Total assessment
1	0	2
2	0	2
3	0	2
4	0	2
5	0	2
6	0	2
7	0	2
**Total**	**0**	**14**

Error coefficient (%) = (0/14)*100 = 0%
Reliability coefficient (%) = 100–0 = 100%

Table 5 Interrater reliability testing between the certified assessor and the PACU nurses

Patient	Number of errors	Total assessment
1	1	11
2	0	11
3	0	11
4	0	11
5	0	11
**Total**	**1**	**55**

Error coefficient (%) = (1/55)*100 = 1.82%
Reliability coefficient (%) = 100–1.82 = 98.18%

Table 6 Thai-version of the Nu-DESC for clinical use

	อาการ (Symptoms)	ระดับความรุนแรงของอาการ (Symptoms Rating)
1	**ความงุนงงสับสน (Disorientation)**<br>การพูดหรือพฤติกรรมที่แสดงว่าไม่รับรู้เวลา หรือสถานที่ หรือบุคคลได้ถูกต้องในสิ่งแวดล้อมขณะนั้น<br>(Verbal or behavioral manifestation of not being oriented to time or place or misperceiving persons in the environment)	☐ 0 ☐ 1 ☐ 2
2	**พฤติกรรมที่ไม่เหมาะสม (Inappropriate behavior)**<br>พฤติกรรมที่ไม่เหมาะสมต่อสถานที่ และ/หรือ ต่อบุคคล เช่น ดึงท่อต่าง ๆ หรือเสื้อผ้า พยายามลงจากเตียงทั้งที่มีข้อห้าม และกรณีอื่นที่คล้ายกัน<br>(Behaviour inappropriate to place and/or for the person; e.g., pulling at tubes or dressings, attempting to get out of bed when that is contraindicated, and the like)	☐ 0 ☐ 1 ☐ 2
3	**การสื่อสารที่ไม่เหมาะสม (Inappropriate communication)**<br>การสื่อสารที่ไม่เหมาะสมต่อสถานที่ และ/ หรือ ต่อบุคคล เช่น การพูดที่ไม่ปะติดปะต่อ ไม่สามารถสื่อความได้ ไม่มีความหมาย หรือ ไม่สามารถเข้าใจได้<br>(Communication inappropriate to place and/or for the person; e.g., incoherence, noncommunicativeness, nonsensical or unintelligible speech)	☐ 0 ☐ 1 ☐ 2
4	**การแปลสิ่งเร้าผิด/ประสาทหลอน (Illusions/Hallucinations)**<br>การเห็นภาพหรือได้ยินสิ่งที่ไม่มีในที่นั้น การเห็นวัตถุบิดเบือนไป<br>(Seeing or hearing things that are not there; distortions of visual objects)	☐ 0 ☐ 1 ☐ 2
5	**สติและการเคลื่อนไหวที่เชื่องช้า (Psychomotor retardation)**<br>การตอบสนองช้า มีการกระทำ/คำพูดที่เกิดโดยทันทีน้อยหรือไม่มีเลย เช่น เมื่อผู้ป่วยถูกกระตุ้น กลับมีการตอบสนองที่ช้า และ/หรือ ไม่มีการตอบสนองต่อตัวกระตุ้น<br>(Delayed responsiveness, few or no spontaneous actions/words; e.g., when the patient is prodded, reaction is deferred and/or the patient is unarousable)	☐ 0 ☐ 1 ☐ 2
	**Total score**	☐ 0 ☐ 1 ☐ ≥ 2

Scoring codes were classified according to severity of symptoms: 0 = no symptom; 1 = mild symptom; 2 = severe symptom. A total score ≥ 2 indicates delirium.
